# Mitogenome recovered from a 19 ^th^ Century holotype by shotgun sequencing supplies a generic name for an orphaned clade of African weakly electric fishes (Osteoglossomorpha, Mormyridae)

**DOI:** 10.3897/zookeys.1129.90287

**Published:** 2022-11-16

**Authors:** John P. Sullivan, Carl D. Hopkins, Stacy Pirro, Rose Peterson, Albert Chakona, Tadiwa I. Mutizwa, Christian Mukweze Mulelenu, Fahad H. Alqahtani, Emmanuel Vreven, Casey B. Dillman

**Affiliations:** 1 National Center for Biotechnology Information, National Library of Medicine, National Institutes of Health, Bethesda, Maryland, USA; 2 Cornell University Museum of Vertebrates, Ithaca, New York, USA; 3 Department of Neurobiology and Behavior, Cornell University, Ithaca, New York, USA; 4 Iridian Genomes, Bethesda, Maryland, USA; 5 The George Washington University, Washington, D.C., USA; 6 NRF-South African Institute for Aquatic Biodiversity, Makhanda, South Africa; 7 Department of Ichthyology and Fisheries Science, Rhodes University, Makhanda, South Africa; 8 Département de Zootechnie, Faculté des Sciences Agronomiques, Université de Kolwezi, Kolwezi, Democratic Republic of the Congo; 9 Département de Gestion des Ressources Naturelles Renouvelables, Unité de recherche en Biodiversité et Exploitation durable des Zones Humides, Université de Lubumbashi, Lubumbashi, Democratic Republic of the Congo; 10 Laboratory of Biodiversity and Evolutionary Genomics, Katholieke Universiteit, Leuven, Belgium; 11 Zoology Department, Ichthyology, Royal Museum for Central Africa, Tervuren, Belgium; 12 National Centre for Bioinformatics, King Abdulaziz City for Science and Technology, Riyadh, Saudi Arabia; 13 Department of Ecology and Evolutionary Biology, Cornell University, Ithaca, New York, USA

**Keywords:** Angolan freshwater fishes, *Heteromormyrus*, *Hippopotamyrus*, historical DNA, mitogenomics, mormyrid, museomics, slender stonebasher

## Abstract

*Heteromormyrus* Steindachner, 1866, a genus of Mormyridae (Teleostei: Osteoglossomorpha), has been monotypic since the description of *Heteromormyruspauciradiatus* (Steindacher, 1866) from a single specimen. No type locality other than “Angola” was given and almost no specimens have been subsequently identified to this species. In order to investigate the relationship of this taxon to fresh specimens collected in Angola and elsewhere, whole genome paired-end sequencing of DNA extracted from the holotype specimen of *Heteromormyruspauciradiatus* was performed and a nearly complete mitogenome assembled from the sequences obtained. Comparison of cytochrome oxidase I and cytochrome *b* sequences from this mitogenome to sequences from recently collected material reveal that *Heteromormyruspauciradiatus* is closely related to specimens identified as *Hippopotamyrusansorgii* (Boulenger, 1905), *Hippopotamyrusszaboi* Kramer, van der Bank & Wink, 2004, *Hippopotamyruslongilateralis* Kramer & Swartz, 2010, as well as to several undescribed forms from subequatorial Africa collectively referred to in the literature as the “*Hippopotamyrusansorgii* species complex” and colloquially known as “slender stonebashers.” Previous molecular phylogenetic work has shown that these species are not close relatives of *Hippopotamyruscastor* Pappenheim, 1906, the type species of genus *Hippopotamyrus* Pappenheim, 1906 from Cameroon, and are thus misclassified. *Hippopotamyrusansorgii* species complex taxa and another species shown to have been misclassified, *Paramormyropstavernei* (Poll, 1972), are placed in genus *Heteromormyrus* and one genetic lineage from the Kwanza and Lucala rivers of Angola are identified as conspecific *Heteromormyruspauciradiatus*. Three additional new combinations and a synonymy in Mormyridae are introduced. The morphological characteristics and geographical distribution of the genus *Heteromormyrus* are reviewed. The electric organ discharges (EODs) of *Heteromormyrus* species are to be treated in a separate study.

## Introduction

Mormyrid fishes are well known for their unusual morphologies, large brains, and ability to generate and sense weak electric pulses for object location and communication (Hopkins 1986; Carlson et al. 2019; Kramer 2021). With more than 230 species placed in 21 genera, Mormyridae is by far the largest family of extant Osteoglossomorpha, among the most speciose families of freshwater fishes endemic to Africa (Froese and Pauly 2022; Sullivan and Lavoué 2022), and along with the otophysan gymnotiforms of the Neotropics one of two great continental radiations of weakly electric teleosts (Moller 1995; Lavoué et al. 2012; Crampton 2019; Ford and Albert 2022).

Despite efforts at taxonomic revision in the pre-molecular era (Géry 1968; Taverne 1971a, b, 1972) the generic classification of Mormyridae is only modestly improved since American ichthyologist George Myers termed it “chaotic” more than sixty years ago (Myers 1960). Over the last quarter century molecular phylogenetics has been instrumental in identifying both natural and artificial genera in this family (Alves-Gomes and Hopkins 1997; Lavoué et al. 2000; Sullivan et al. 2000, 2016; Levin and Golubtsov 2017; Peterson et al. 2022), but changes to mormyrid classification have not always followed hard on the heels of these discoveries. Here we describe progress clarifying generic concepts in one small segment of the Mormyridae made possible by sequencing DNA from a 19^th^ Century holotype specimen.

The “*Hippopotamyrusansorgii* species complex” (HaSC) is an informal term used to denote *Hippopotamyrusansorgii* (Boulenger, 1905) plus three more described and additional undescribed species of mormyrid weakly electric fishes known in anglophone southern Africa as “slender stonebashers” (Kramer et al. 2004; Chakona et al. 2018; Mutizwa et al. 2021). Previous analyses of DNA sequence data have shown that these species, while a monophyletic group, are not close relatives of *Hippopotamyruscastor* Pappenheim, 1906, the type species of *Hippopotamyrus* Pappenheim, 1906 from Cameroon, but are instead the sister group to a very large clade of mostly Congo Basin species placed in genera *Marcusenius* Gill, 1862, *Gnathonemus* Gill, 1863, *Campylomormyrus* Bleeker, 1874, *Genyomyrus* Boulenger, 1898, and *Cyphomyrus* Myers, 1960 (Sullivan et al. 2016; Peterson et al. 2022). The recent phylogenomic study of Peterson et al. (2022) additionally showed that two forms from opposite ends of the Congo Basin belong to the *Ha*SC: *Paramormyropstavernei* (Poll, 1972) of the upper Lualaba and Lufira rivers of southeastern D.R. Congo and an undescribed species collected in the Inkisi River in southwestern D.R. Congo.

Losing their parent taxon (*Hippopotamyrus*) renders these species taxonomic orphans. Given this clade’s phylogenetic position within Mormyridae it is not obvious which other genus as currently defined could accept them. Before considering introduction of a new generic name, we needed first to determine whether an available name with priority exists. We wondered if the natural home for these orphaned species might be *Heteromormyrus* Steindachner, 1866, coincidently the single valid mormyrid genus missing from published molecular datasets.

Franz Steindachner described Mormyrus (Heteromormyrus) pauciradiatus from a single specimen, catalogued in the Naturhistorisches Museum Vienna as NMW 22417 (Steindachner 1866, Fig. 1). The type locality was given only as “Angola.” Taverne (1972) elevated the subgeneric name *Heteromormyrus* to genus, but included no additional species under it. *Heteromormyrus* has remained monotypic and the non-type holdings of *Heteromormyruspauciradiatus* (Steindachner, 1866) in public collections consist of only a single specimen, BMNH 1910.11.28.44 from Cabiri on the Bengo River of Angola, identified by George Boulenger more than one century ago. While appearing in published faunal lists and databases as a valid species (e.g., Gosse 1984; Skelton 2019; Fricke et al. 2022), *H.pauciradiatus* is essentially a taxon inquirendum, a name known only from the original description and of doubtful application to any recently collected specimens.

**Figure 1.**
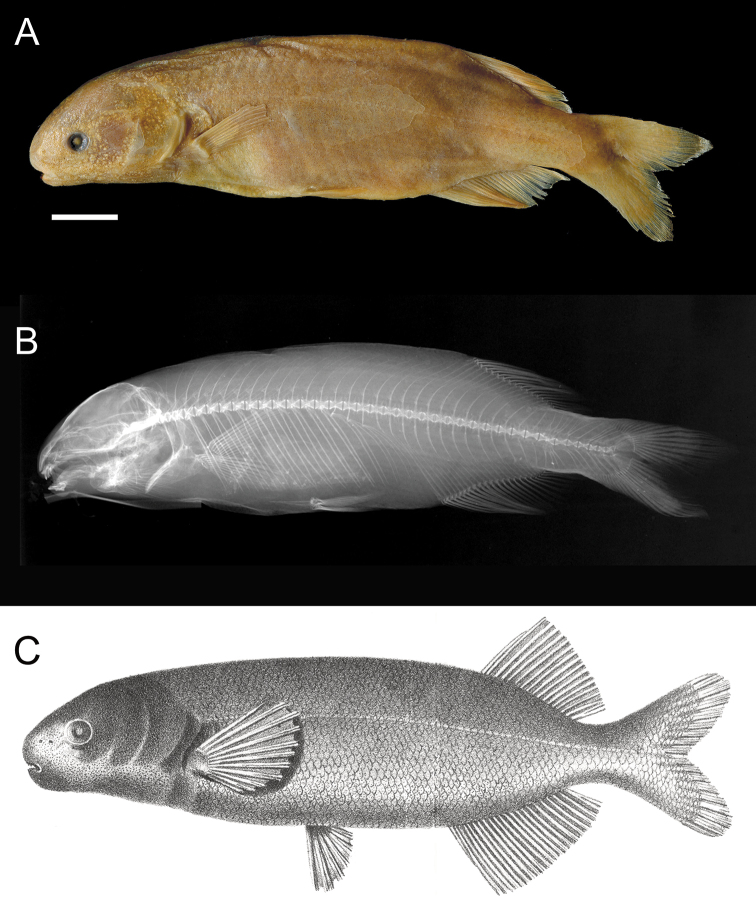
*Heteromormyruspauciradiatus* (Steindacher, 1866) **A** photo **B** radiograph **C** drawing of holotype NMW 22417. Scale bar: 1 cm. Photo- and radiograph courtesy of Naturhistorisches Museum Vienna, drawing from Steindachner (1866).

Reticence of taxonomists to use either this specific or generic name during the past 150 years may be due to the holotype’s unusually short caudal peduncle that places its morphometric ratios outside the range of most other specimens as well as its imprecise type locality. Nonetheless, we suspected *H.pauciradiatus* might be related to the species of the *Ha*SC both because of general phenotypic similarity and its geographic provenance: we know the freshwaters of Angola to be particularly rich in forms belonging to this mormyrid clade, many still undescribed (Mutizwa et al. 2021). If indeed *H.pauciradiatus* and the *Ha*SC constitute a monophyletic group within Mormyridae, the former could provide the generic name the latter require.

Without fresh specimens identified as *H.pauciradiatus* we were prevented from exploring this question using molecular phylogenetics. However, because this type specimen appears to have been originally preserved in ethanol and never fixed in formalin, we thought it might yield DNA. We were encouraged that efforts at PCR-based Sanger sequencing of other Steindachner types from the same era have been successful (Silva et al. 2019) but wished to explore the possibility that high throughput, whole genome short-read or “shotgun” sequencing would yield more sequence with less effort, as suggested by recent studies that have used this methodology to sequence old type specimens in other vertebrate groups (McGuire et al. 2018; Kehlmaier et al. 2019; Reyes-Velasco et al. 2021; Roos et al. 2021).

In contrast to *H.pauciradiatus*, many specimens have been identified (rightly or wrongly) to the taxon *Hippopotamyrusansorgii* (Boulenger, 1905) during the past 117 years, although it is also a species with an uncertain type locality. However, in a recent study Mutizwa et al. (2021) examined the field notes of the collector of *H.ansorgii’s* two syntypes and concluded that they must have been taken in the Kwanza River basin of Angola, a system in which the same authors identified five distinct mitochondrial lineages of the *Ha*SC, associated with different morphotypes. Mutizwa et al. (2021) informally named these K1 through K5 without determining which if any of them correspond to *H.ansorgii* sensu stricto, or if one of these might correspond to the odd-looking holotype of *H.pauciradiatus*, a fish that may or may not have come from the Kwanza.

## Materials and methods

The curator of the Naturhistorisches Museum Vienna supplied us with right-side gill arch and gill filament tissue as well as a right-side pectoral fin clip from the *Heteromormyruspauciradiatus* holotype, NMW 22417. Specimen measurements and counts performed for this study follow Kramer and Swartz (2010). Institutional abbreviations follow Sabaj (2020). Other abbreviations used: standard length = **SL**, electric organ discharge = **EOD**, base pairs = **bp**.

### DNA extraction, library preparation, and sequencing

DNA extraction, Illumina platform library preparation and whole-genome short-read sequencing was performed by the GeneWiz Corporation of South Plainfield, New Jersey, USA and were conducted separately for the fin clip and the gill arch tissue samples.

Genomic DNA was extracted with a Qiagen DNeasy Kit with the following three modifications to the manufacturer-recommended procedure: (1) wide-bore pipette tips were used to minimize DNA damage by shearing; (2) the proteinase K digestion was performed for 15 minutes only; (3) elution consisted of three repeats of adding 10 µl of 0.1× TE warmed to 37 °C to the spin column, incubated for 10 min at 37 °C before collection by centrifugation.

For library preparation the Illumina TruSeq library kit was used with these modifications of the manufacturer’s recommended protocol for the degraded sample: no size selection of DNA was performed and half the recommended amounts of all reagents were used.

The constructed libraries were sequenced as 150 bp paired ends on the Illumina HiSeq X Ten platform, multiplexed five samples per run. The reads were deposited into NCBI’s Sequence Read Archive (**SRA**) under the accession number SRX7700131.

### Mitogenome reconstruction

For a separate project on Osteoglossomorpha phylogeny Peterson et al. (2022) sequenced 40 mormyrid genomes on the Illumina platform from tissue samples originally taken from fresh specimens. Among these genomes is one from the holotype specimen of *Hippopotamyruslongilateralis* Kramer & Swartz, 2010 (SAIAB 78793), a species belonging to the *Ha*SC, a cytochrome *b* sequence from which we determined had high similarity to one we initially recovered from the *H.pauciradiatus* type specimen NMW 22417 sequence fragments (see Results below).

From the SRA of the *H.longilateralis* specimen, SRX5986274, we generated a complete mitogenome using the SMART2 (Statistical Mitogenomes Assembly with RepeaTs) pipeline (Alqahtani and Măndoiu 2020a, b). Read pairs of mitochondrial origin in this SRA are 0.2% of the total reads and the average read depth of this mitogenome is 5,450×. Subsequently we annotated the genome using MITOS (Bernt et al. 2013) and submitted it to NCBI/GenBank where it is published under accession MZ151890. This mitogenome reflects the standard complement of elements in the standard order for vertebrates: 13 protein-coding genes, 22 tRNA genes, two rRNA genes, and one control region or “d-loop,” with the ND6 gene and eight tRNA genes encoded on the L-strand (Satoh et al. 2016).

The same SMART2 pipeline was unable to reconstruct the mitogenome from the SRA for the *H.pauciradiatus* holotype NMW 22417, likely due to the much lower quantity of data sequenced from the highly degraded DNA present in the extraction.

Alternatively, we used the entire *H.longilateralis* mitogenome sequence as a query sequence in a BLAST (Basic Local Alignment Search Tool) search (Standard Nucelotide BLAST) against the SRA library obtained from NMW 22417 (SRX7700131). We conducted two such searches: one using the setting for “highly similar sequences (megablast)” and the other with the setting “somewhat similar sequences (blastn)” with maximum target sequences set to 5000, max matches in a query range set to 50, and all other settings at default values. From the results page of each search, using the alignment view “query-anchored with dots for identities” and setting line length to 150, we downloaded the alignments as text files. We reconstructed the sequence of the holotype in a text editor by changing bases in the top (query) sequence where necessary to match those in the high similarity aligned sequences, using ambiguity codes and “N” for missing sites where necessary. (Assembling a long, new sequence of interest from short read sequence data in NCBI’s SRA using a query sequence from a related species in NCBI’s Web BLAST tool is demonstrated in an online video (Sullivan 2022) and is here termed “SRA BLASTing.”) To check the quality of this mitogenome, we analyzed and annotated it with MitoFish MitoAnnotator version 3.61 (Iwasaki et al. 2013). Using the Python script MitoFish2tbl (Hahn 2020) we converted the annotation file produced by MitoAnnotator to the “.tbl” format needed for GenBank submission.

### Phylogenetic methods

We used cytochrome *b* (Cyt-b) and cytochrome oxidase I (COI) sequences from the reconstructed mitogenome of *Heteromormyruspauciradiatus* holotype NMW 22417 in two separate phylogenetic analyses that included previously unpublished sequences of both markers from a specimen of *Paramormyropstavernei* (Poll, 1972), RMCA Vert 2018-032-P-0047, from the upper Lufira River in D.R. Congo and an undescribed *Ha*SC species from the Inkisi River in D.R. Congo (AMNH 247102). The latter two specimens had been included in the recent phylogenomic study of Peterson et al. (2022).

For the Cyt-*b* analysis we began with the multi-locus alignment used by Sullivan et al. (2016) of 4209 bases of mitochondrial cytochrome *b*, 12S, 16S, and nuclear *rag2* and *rps7* intron that, with the exception of *Heteromormyrus*, included representatives of all nominal genera from the Mormyrinae. Based on results of previous phylogenetic studies of Mormyroidea (Sullivan et al. 2000; Peterson et al. 2022) the tree was rooted with *Myomyrusmacrops*. To this alignment we added the Cyt-*b* sequences from NMW 22417, the *P.tavernei* and Inkisi River specimens, plus previously published Cyt-*b* sequences of *Ha*SC species and populations from Kramer et al. (2004), Kramer and Swartz (2010), and Mutizwa et al. (2021) as well as new sequences from *H.szaboi*-like forms from the Kabompo River, a large left-bank affluent of the upper Zambezi in Zambia. As analyzed, this dataset included sequences from 113 individuals. Missing data was coded as “?”.

The separate COI alignment included sequences from the *H.pauciradiatus* holotype NMW 22417, the *P.tavernei* and the Inkisi River specimens, plus all COI sequences from *Ha*SC species publicly available in BOLD and GenBank. The analysis included 96 individuals and was rooted with a sequence from *Marcuseniuscyprinoides* from the BOLD database. This outgroup taxon was chosen as a member of the sister group to the *Heteromormyrus* clade based on the results of Peterson et al. (2022).

All ingroup specimens used in both analyses are listed in Suppl. material 1.

We inferred these two phylogenetic trees using the maximum likelihood optimality criterion in RAxML HPC v. 8.2.12 (Stamatakis 2014) on the CIPRES Science Gateway supercomputing cluster (Miller et al. 2010). In the multilocus alignment, we assigned separate partitions to each codon position of each coding gene (Cyt-*b*, *rag2*) as well as to each of the non-coding genes (12S, 16S, *rps7* intron) for a total of nine partitions. Likewise, we partitioned the COI dataset by codon position. We determined the optimal model of molecular evolution for each partition using the Bayesian information criterion in Partition Finder2 (Lanfear et al. 2012), constrained to select from models available in RAxML. The GTR+gamma or GTR+gamma+I models were selected by Partition Finder as optimal for all partitions of in both analyses. As calculation of invariant sites (I) is discouraged by the author of RAxML, we used separate GTR+gamma (GTRGAMMA) evolutionary models for all partitions and performed a non-parametric bootstrap analysis using the faster GTRCAT model to estimate support for nodes. Bootstrapping was auto-terminated using the autoMRE criterion. All other settings were left at their default values.

## Results

### Holotype mitogenome assembly

Sequencing the DNA extraction from the holotype fin clip yielded no data. Sequencing the DNA extraction from the gill arch tissue yielded 1.3 gigabases of paired-end sequences, available on NCBI’s Sequence Read Archive under accession SRX7700131. For comparison, Illumina sequencing of 40 other mormyrid taxa from tissues sampled within the past 20 years (preserved in 95% ethanol or buffer) yielded between 21 and 32 gigabases of sequence data.

To ascertain whether mitochondrial sequences were present in the recovered data we used a 1060 bp Cyt-*b* sequence from a specimen identified as *H.ansorgii* accessioned in the GenBank Nucleotide database (AY236991) as a query sequence in a BLAST search against the SRA data in SRX7700131. BLAST hits of high similarity were matched across nearly the entire query sequence. On the BLAST search result page we set the alignment view to “query-anchored with dots for identities” with a line length of 150 characters and downloaded the results as text. We reconstructed all but 23 bases of the 1060 bp fragment from NMW 22417 in a text editor by changing bases in the top (query) sequence where necessary to match those in the high similarity aligned sequenced fragments.

We BLASTed (using megablast) this reconstructed Cyt-*b* sequence against the entire NCBI Nucleotide archive and found highest similarity (94.6% to 96.5%) to sequences identified as *Hippopotamyrus* sp., *H.ansorgii*, *H.szaboi*, and *H.longilateralis*.

To test whether our reconstructed sequence may have been affected by the choice of query sequence we repeated the process using two different mormyrid Cyt-*b* sequences from GenBank as queries: *Marcuseniusmoorii*AF201595 and *Paramormyropskingsleyae*AF477422. In both cases, the sequences we reconstructed for NMW 22417 matched the sequence reconstructed using the *H.ansorgii* query base-for-base, although many more differences were observed between the SRA sequences and these query sequences.

Using this method of “SRA BLASTing” and downloading the resulting text file (Sullivan, 2022), we reconstructed the mitogenome of the *Heteromormyruspauciradiatus* holotype using the complete mitogenome of *H.longilateralis* (GenBank accession MZ151890) as query sequence. Short stretches of bases in the query sequence that found no matches in the BLAST search of the SRA were coded as Ns in the reconstructed sequence.

We found 100 cytosine (C) sites in the query sequence at which aligned reads from holotype NMW 22417 are both C and thymine (T), producing a Y ambiguity code in the inferred sequence. We noted a smaller but significant number (24) of ambiguous calls at sites where a guanine (G) in the query sequence is matched to holotype sequences containing both G and adenine (A) that we coded with an R ambiguity code. Sites where Ts and As in the query sequence produced ambiguity codes in the reconstructed sequence were much less common. This observation is consistent with a known consequence of sequencing template DNA that has been subject to hydrolytic deamination of cytosine to uracil (U), a naturally occurring process as DNA degrades in an aqueous solution. In these cases the polymerase incorporates an A across from each deaminated U site in the template DNA fragments and then in turn a T across from each A on the complementary strands, producing apparent G → A and C → T substitutions (Dabney et al. 2013).

**Figure 2.**
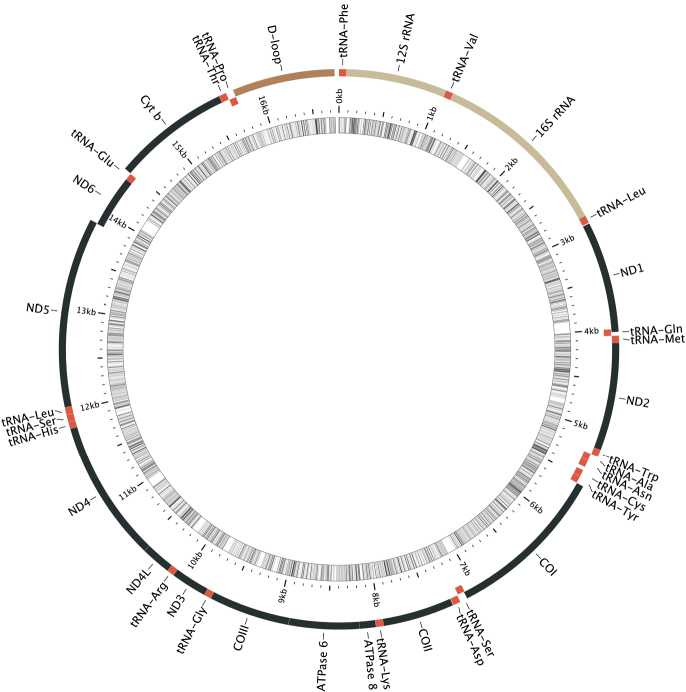
Visual representation of 16750 base-pair mitochondrial genome of *Heteromormyruspauciradiatus* holotype specimen NMW 22417 as reconstructed from 150 base-pair, paired-end sequence data. Image produced from MitoFish MitoAnnotator v. 3.63. Coding genes black, non-coding regions red (tRNA genes), gold (ribosomal RNA genes) and brown (control region or D-loop). Genes transcribed on L-strand indented. Innermost circle represents percent GC per five base-pair segment, darker = higher; white areas indicate missing data. Because no sequences were recovered for the short tRNA-Ile gene, it appears absent from its normal position between genes ND1 and tRNA-Gln. Annotated mitogenome available in NCBI GenBank as accession ON533765.

The final mitogenome consists of 16750 bp with 781 sites (4.6%) coded as N for missing, 100 coded as Y, 24 as R, 11 as M (A or C) and four as W (A or T). (In calculating the number of missing bases scored as N in the reconstructed *H.pauciradiatus* mitogenome we made the assumption that missing sequence was equal in length to the *H.longilateralis* query sequence. There are likely to be small length differences between the two mitogenomes in non-coding regions, hence the reported total length of the holotype mitogenome is inexact.) The circular genome as reconstructed by the MitoFish MitoAnnotator is shown in Fig. 2. The file from which we reconstructed the mitogenome of the holotype SRA is provided in Suppl. material 2. The annotated mitogenome was submitted to NCBI Nucleotide Database (GenBank) and is published as accession ON533765.

Using the same method, we attempted to reconstruct nuclear markers *rps7* and *rag2* from the holotype SRA using mormyrid sequences available in GenBank as query sequences, but BLAST searches found no significant matches. We suspect our greater success recovering mitochondrial sequences has to do with the far higher cellular copy number of mitochondrial versus nuclear genomes, enhancing the probability of persistence of some long DNA fragments of mitochondrial origin in highly degraded templates (Merheb et al. 2019).

Accession numbers and their GenSeq status (Chakrabarty et al. 2013) for sequences and genomes generated for this study given in Table 1.

**Table 1.** Accession numbers and GenSeq status for DNA sequences and genomes generated for this study, archived in NCBI GenBank and the NCBI Sequence Read Archive (SRA). WG = whole genome (unassembled Illumina paired-end reads).

Species	Voucher Catalog No.	Type Status	Locus/Loci	Sequence Read Achive	GenBank Accession(s)	GenSeq status
*Heteromormyruspauciradiatus*	NMW 22417	holotype	WG, mitogenome	SRX7700131	ON533765	genseq-1 mitogenome
*Heteromormyruslongilateralis*	SAIAB 78793	holotype	WG, mitogenome	SRX5986274	MZ151890	genseq-1 mitogenome
*Heteromormyrustavernei*	RMCA Vert 2018-032-P-0047	nontype	COI, Cyt-*b*	–	ON843622, ON858019	genseq-4 COI, genseq-4 CytB
*Heteromormyrus* sp. Inkisi River	AMNH 247102	nontype	COI, Cyt-*b*	–	ON843623, ON858020	–
Heteromormyrusaff.szaboi Kabompo EODtype1	SAIAB 210091/KW12-AT3408/SB8355	nontype	Cyt-*b*	–	ON088276	–
Heteromormyrusaff.szaboi Kabompo EODtype1	SAIAB 210127/KW12-AT2748/SB8356	nontype	Cyt-*b*	–	ON088277	–
Heteromormyrusaff.szaboi Kabompo EODtype2	SAIAB 210191/KW12-AT3411/SB8357	nontype	Cyt-*b*	–	ON088278	–
Heteromormyrusaff.szaboi Kabompo EODtype1	SAIAB 210191/KW12-AT1012/SB8358	nontype	Cyt-*b*	–	ON088279	–
Heteromormyrusaff.szaboi Kabompo EODtype1	SAIAB 210191/KW12-AT2645/SB8359	nontype	Cyt-*b*	–	ON088280	–
Heteromormyrusaff.szaboi Kabompo EODtype1	SAIAB 210191/KW12-AT4962/SB8360	nontype	Cyt-*b*	–	ON088281	–
Heteromormyrusaff.szaboi Kabompo EODtype2	SAIAB 210191/KW12-AT2664/SB8361	nontype	Cyt-*b*	–	ON088282	–
Heteromormyrusaff.szaboi Kabompo no EOD	SAIAB 210234/KW12-AT4937/SB8362	nontype	Cyt-*b*	–	ON088283	–
Heteromormyrusaff.szaboi Kabompo no EOD	SAIAB 210234/KW12-AT2000/SB8363	nontype	Cyt-*b*	–	ON088284	–
Heteromormyrusaff.szaboi Kabompo no EOD	SAIAB 210243/KW12-AT4914/SB8364	nontype	Cyt-*b*	–	ON088285	–
Heteromormyrusaff.szaboi Kabompo no EOD	SAIAB 210257/KW12-AT4203/SB8365	nontype	Cyt-*b*	–	ON088286	–
Heteromormyrusaff.szaboi Kabompo no EOD	SAIAB 210272/KW12-AT4984/SB8366	nontype	Cyt-*b*	–	ON088287	–
Heteromormyrusaff.szaboi Kabompo EODtype1	SAIAB 210149/KW12-AT912/SB8367	nontype	Cyt-*b*	–	ON088288	–

### Phylogenetic analyses

**Figure 3.**
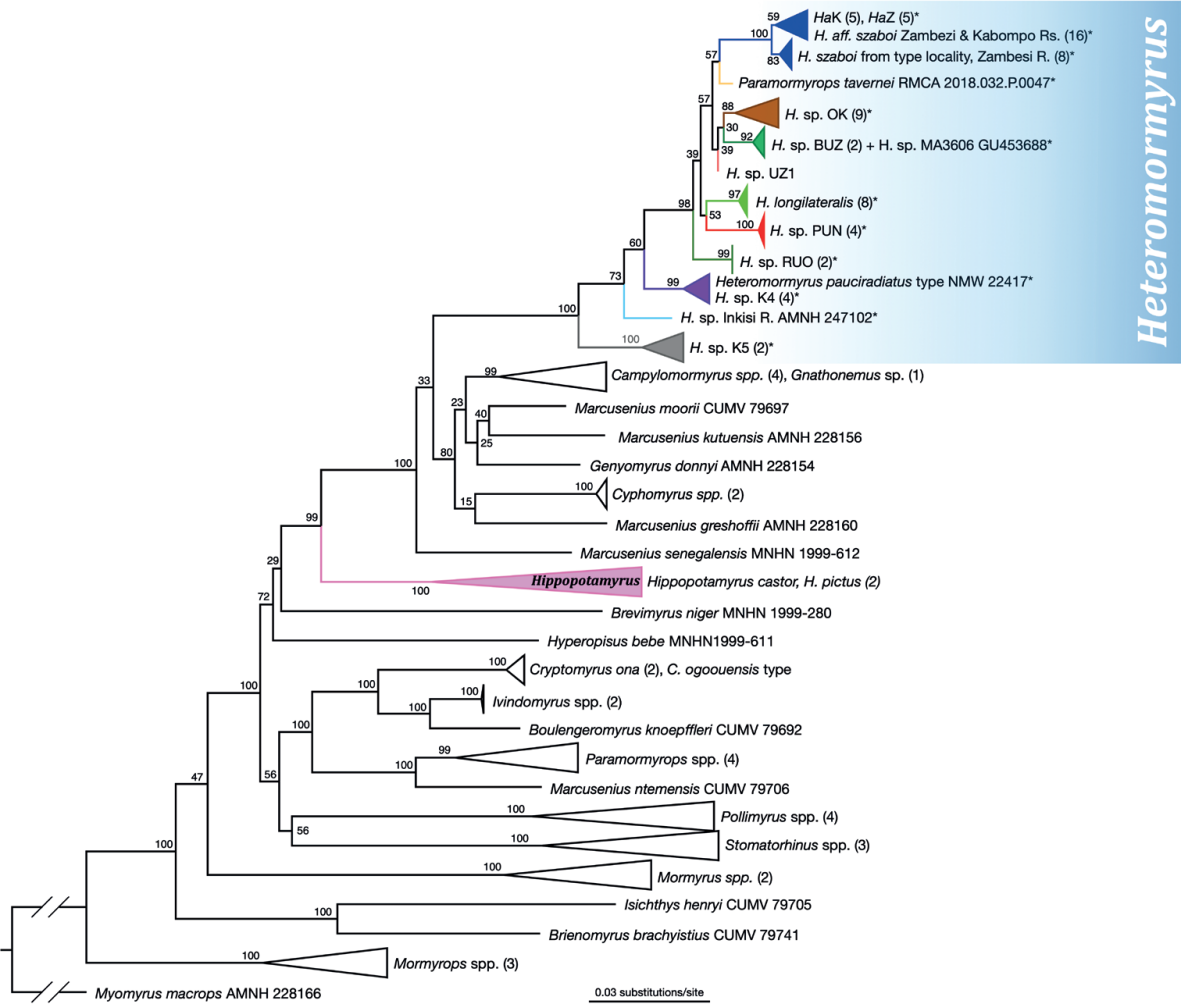
Phylogenetic position of *Heteromormyruspauciradiatus* and other taxa of interest within subfamily Mormyrinae, inferred from cytochrome *b* and other markers. Maximum likelihood phylogenetic tree calculated in RAxML of 113 mormyrin OTUs, including all nominal mormyrin genera, from alignment of 4209 bases of mitochondrial cytochrome *b*, 12S, 16S and nuclear *rag2* and *rps7* intron. Rooted with *Myomyrusmacrops*. Dataset is from Sullivan et al. (2016) to which was added all publicly available *Hippopotamyrusansorgii* species complex cytochrome *b* sequences with the addition of sequences from *Heteromormyruspauciradiatus* holotype NMW 22417, the *Hippopotamyruslongilateralis* holotype, *Paramormyropstavernei*, an undescribed species from the Inkisi River in D.R. Congo and several sequences from H.aff.szaboi from the Zambezi and Kabompo rivers, Zambia. Asterisks indicate taxa represented by cytochrome *b* sequences only. Genus *Hippopotamyrus* is shown to be diphyletic with type species *Hippopotamyruscastor* clustering with *Hippopotamyruspictus* separately from the *Hippopotamyrusansorgii* species complex clade containing the holotype of *Heteromormyruspauciradiatus*. The latter clade we reclassify as *Heteromormyrus*. Full tree with nodes uncollapsed available as Suppl. material 3. OTU names and colors match those used in Mutizwa et al. (2021).

**Figure 4.**
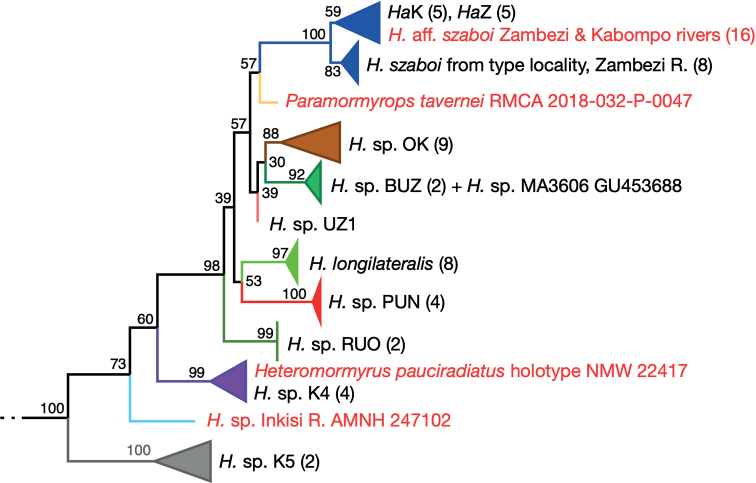
Enlargement of the newly recognized *Heteromormyrus* clade (including species and informally named entities formerly said to belong to the *Hippopotamyrusansorgii* species complex) from the full tree in Fig. 3. New taxa added for this study indicated by red typeface. *Heteromormyruspauciradiatus* holotype sequence clusters with sequences from the K4 lineage. Another misclassified taxon, *Paramormyropstavernei*, and an undescribed taxon from the Inkisi River of the D.R. Congo appear within this clade as well, with strong bootstrap support.

The tree produced from the phylogenetic analysis of the Cyt-*b* sequence data added to the Sullivan et al. (2016) dataset (Figs 3, 4; tree with all terminals shown in Suppl. material 3) demonstrates that the holotype of *Heteromormyruspauciradiatus* as well as a specimen of *Paramormyropstavernei* and an undescribed form from the Inkisi River of D.R. Congo belong to a clade within Mormyrinae containing species heretofore placed in genus *Hippopotamyrus* (*H.szaboi*, *H.longilateralis*) along with several unidentified or undescribed forms from subequatorial Africa, the group collectively referred to as the *Ha*SC in recent literature (Kramer et al. 2004; Kramer and Swartz 2010; Mutizwa et al. 2021).

In this tree the *H.pauciradiatus* holotype sequence falls within a cluster of three haplotypes from four specimens of the K4 mitochondrial lineage identified in Mutizwa et al. (2021) and with an allowance for the five bases coded “N” and two coded “Y” has an identical Cyt-*b* sequence to two of these, SAIAB 85203 (MW600881) and SAIAB 85209 (MW600880). Both of these specimens were collected in 2009 from the same site on the Lucala River in Angola with coordinates 9°25'30.0"S, 14°42'0.0"E. The remaining two K4 specimens are from downstream sites on the Lucala.

The Cyt-*b* sequence from *Paramormyropstavernei* places it as sister lineage to a clade containing *H.szaboi* and related forms while the sequence from the Inkisi River specimen places it in a position subtending all other lineages within the *Ha*SC clade with the exception of the K5 lineage that is sister group to the rest.

This tree recapitulates the finding reported in Sullivan et al. (2016) and in Peterson et al. (2022) that the *Ha*SC clade is not monophyletic with *Hippopotamyruscastor* Pappenheim, 1906 from Cameroon, the type species of genus *Hippopotamyrus* and supports the conclusion that the *Ha*SC taxa are misclassified in that genus (Fig. 3).

**Figure 5.**
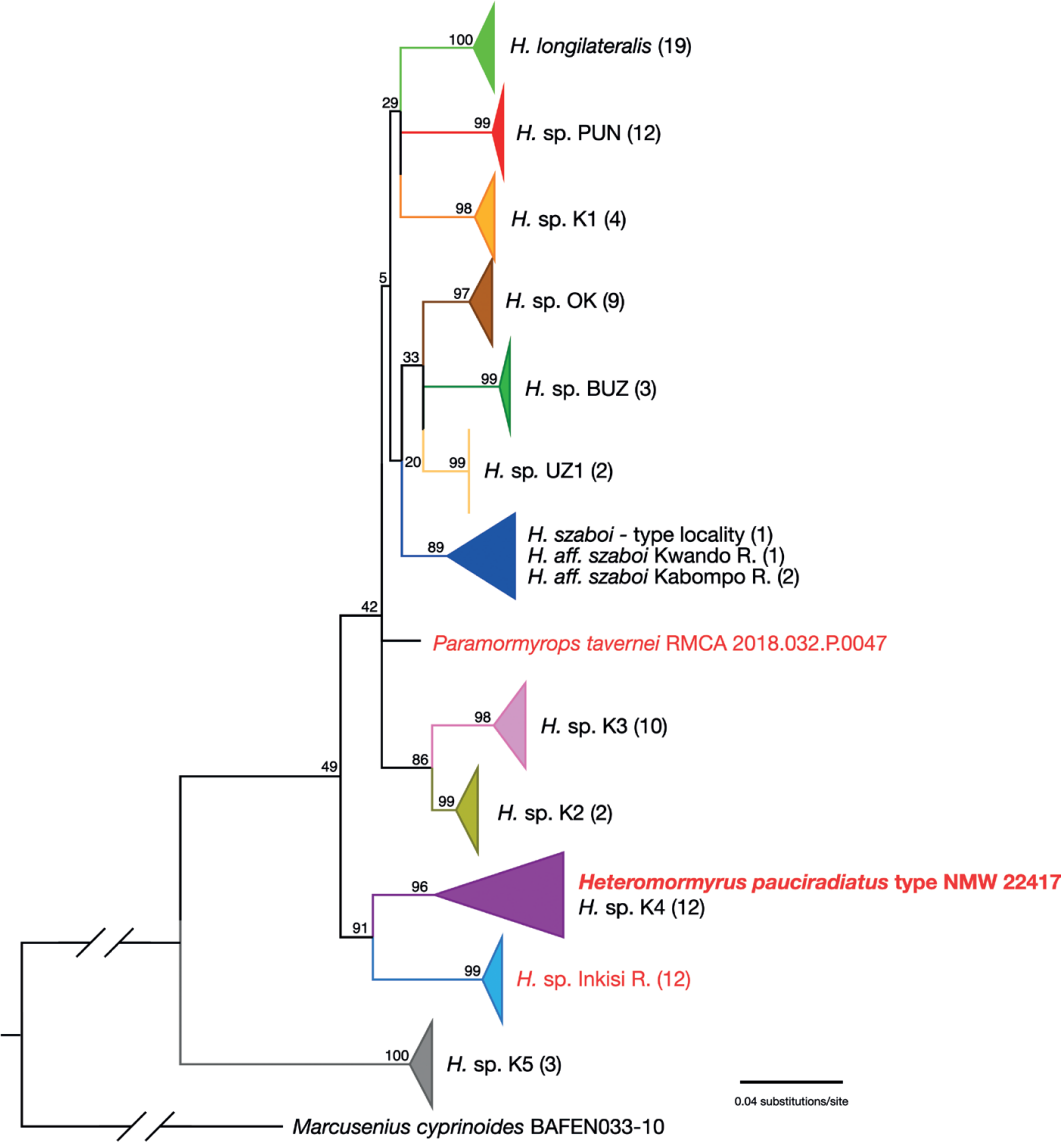
Maximum likelihood phylogenetic tree calculated in RAxML for 96 *Hippopotamyrusansorgii* species complex COI sequences from the Barcode of Life Database (BOLD) with the addition of sequences from the holotype of *Heteromormyruspauciradiatus* NMW 22417 (bold red type), *Hippopotamyruslongilateralis* holotype, *Paramormyropstavernei* (red type) and an undescribed species from the Inkisi River in D.R. Congo (red type). Rooted with *Marcuseniuscyprinoides*. Bootstrap values at selected nodes. Organismal names reproduced as they appear in BOLD. Colors of species/clades match those used in Mutizwa et al. (2021). Full tree with nodes uncollapsed in Suppl. material 4.

The tree produced from the phylogenetic analysis of the COI sequence data (Fig. 5; tree with all terminals shown in Suppl. material 4) again shows the sequence from the *Heteromormyruspauciradiatus* holotype nested within the K4 lineage clade (six unique haplotypes from 12 specimens). The holotype COI sequence is identical to one deposited in the BOLD database with sequence code SAFW518-09. This specimen, now catalogued as SAIAB 85120, was collected in 2008 at a site on the upper Lucala River just above the 105-meter-high, 400-meter-wide Kalandula Falls (09°4'26"S, 16°0'0"E). All but one of the K4 sequences are from specimens collected in the Lucala River, the northeastern tributary of the Kwanza. The remaining one, MW600858 from SAIAB 84726, is from the confluence of the Kawa and the Kwanza rivers in the lower Kwanza Basin, 09°10'17"S, 13°22'5"E. In this analysis, the undescribed species from the Inkisi River forms the sister lineage to the K4 clade and *P.tavernei* is unresolved within a large clade containing *H.szaboi*, *H.longilateralis*, K1–K3, and others.

In both analyses, bootstrap values indicate strong support for the inclusion of the *H.pauciradiatus* holotype sequences within the lineage called K4 by Mutizwa et al. (2021) and for inclusion of *P.tavernei* and the undescribed species from the Inkisi River within the larger *Ha*SC clade, itself well supported as a monophyletic group. A single topology of relationships among species and named clades within this group is not well supported by these data.

### Taxonomy

Based on the phylogenetic results, we regroup six valid species into genus *Heteromormyrus* Steindachner, 1866, a name originally introduced as a subgenus of *Mormyrus* Linnaeus, 1758, but recognized by Taverne (1972) and subsequent authors (Poll and Gosse 1995; Fricke et al. 2022) as a monotypic genus. *Heteromormyrus* has priority as the oldest available generic name with its type species within the clade formed by these species. The taxon indicated by the asterisk below is unsequenced and provisionally placed in *Heteromormyrus* pending study of fresh collections. For simplicity, only the original name combination and first use of subsequent combinations in the literature are shown beneath the name combination recognized here.

#### Family Mormyridae Bonaparte, 1831

**Subfamily Mormyrinae Taverne, 1972**

##### Genus Heteromormyrus

Taxon classificationAnimaliaOsteoglossiformesMormyridae

Steindachner, 1866

2440F06A-A681-52AE-A7A5-EF58998E476B

Heteromormyrus Steindachner, 1866: 765 [as subgenus of Mormyrus; elevated to genus by Taverne 1972: 168; type species = Mormyrus (Heteromormyrus) pauciradiatus Steindachner, 1866, by original monotypy].

#### Species included

##### Heteromormyrus pauciradiatus

Taxon classificationAnimaliaOsteoglossiformesMormyridae

(Steindacher, 1866)

C5A54A6A-6736-5874-928D-E5BD96A8781D

Mormyrus (Heteromormyrus) pauciradiatus Steindachner, 1866: 765, pl. 13, fig. 2.
Marcusenius pauciradiatus (Steindachner) [new combination by Boulenger 1898: 795]
Marcusenius (Heteromormyrus) pauciradiatus (Steindachner) [new subgeneric combination by Géry 1968: 76].
Pollimyrus pauciradiatus (Steindachner) [new combination by Taverne 1971a: 105].
Heteromormyrus pauciradiatus (Steindacher) [new combination by Taverne 1972: 168].

##### Heteromormyrus ansorgii

Taxon classificationAnimaliaOsteoglossiformesMormyridae

(Boulenger, 1905) comb. nov.

3F27F250-A333-50CC-B45F-68166ADA71F8

Marcusenius ansorgii Boulenger, 1905a: 457.
Hippopotamyrus ansorgii (Boulenger) [new combination by Taverne 1971a: 104].

##### Heteromormyrus pappenheimi

Taxon classificationAnimaliaOsteoglossiformesMormyridae

(Boulenger, 1910), comb. nov.*

FBF88C01-AD48-5854-9488-FC169BAEF6EA

Marcusenius pappenheimi Boulenger, 1910: 540.
Hippopotamyrus pappenheimi (Boulenger) [new combination by Taverne 1971a: 104].

##### Heteromormyrus tavernei

Taxon classificationAnimaliaOsteoglossiformesMormyridae

(Poll, 1972) comb. nov.

0A827021-93CE-52B4-8129-7C568E456ED1

Brienomyrus tavernei Poll, 1972: 166, fig. 2.
Paramormyrops tavernei (Poll) [new combination by Rich et al. 2017: 626].

##### Heteromormyrus szaboi

Taxon classificationAnimaliaOsteoglossiformesMormyridae

(Kramer, van der Bank & Wink, 2004) comb. nov.

762169C8-0A0A-5EE3-8110-88A16D83B910

Hippopotamyrus szaboi Kramer, van der Bank & Wink, 2004: 6, fig. 1A, B.

##### Heteromormyrus longilateralis

Taxon classificationAnimaliaOsteoglossiformesMormyridae

(Kramer & Swartz, 2010) comb. nov.

9446EE9F-3366-5E81-AD70-FC33FB41D1C7

Hippopotamyrus longilateralis Kramer & Swartz, 2010: 2231, fig. 1A.

**Figure 6.**
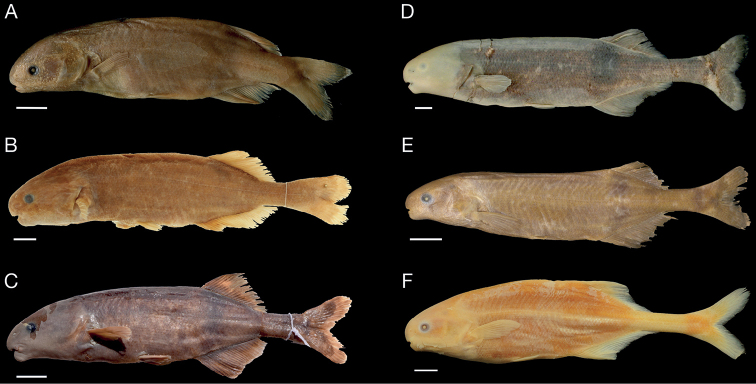
Images of types of *Heteromormyrus* species in left-side lateral view. **A***Heteromormyruspauciradiatus* (Steindacher, 1866), holotype, NMW 22417, Angola, 108 mm TL, photograph Naturhistorisches Museum Vienna **B***Heteromormyrustavernei* (Poll, 1972), holotype, MRAC 79-1-P-137, Masombwe, Kipepe River, tributary of Tumbwe River, Congo River basin, Democratic Republic of the Congo, 139 mm SL; photograph T. Nève, RMCA **C***Heteromormyrusszaboi* (Kramer, van der Bank & Wink, 2004), holotype, SAIAB 67143, upper Zambezi River, Katima Mulilo, 17°29'30"S, 24°16'18"E. 94 mm SL; photograph A. Chakona **D***Heteromormyruslongilateralis* (Kramer & Swartz, 2010), holotype, SAIAB 78793, above Epupa Falls, Kunene River, on the Namibian/Angolan border, 17°00'07"S, 013°14'57"E; 187 mm SL; photograph Kramer & Swartz **E***Heteromormyrusansorgii* (Boulenger, 1905a), syntype BMNH 1905.5.29.62; between Benguella and Bihé [Benguela and Bié], Angola; 96.9 mm SL; photograph J.P. Sullivan **F***Heteromormyruspappenheimi* (Boulenger, 1910), syntype ANSP 37971, Kwanza River at Cunga [= Cabala], Angola, 134 mm SL, right side reflected to face left; photograph K. Luckenbill, ANSP. Scale bars: 1 cm.

Images of type specimens of these species are shown in Fig. 6. Three additional new combinations and a synonymy in Mormyridae are introduced in the Discussion section below.

## Discussion

A more complete history of the generic name *Heteromormyrus* is as follows. As related above, Steindachner (1866) introduced the name as a subgenus of *Mormyrus* for his new species Mormyrus (Heteromormyrus) pauciradiatus, based on the single specimen NMW 22417, the subject of this paper. Boulenger (1899) placed this species under *Marcusenius* Gill and subsequent authors treated this species as *Marcuseniuspauciradiatus* (e.g., Ladiges 1964; Poll 1967) with Géry (1968) recognizing *Heteromormyrus* as a subgenus of *Marcusenius*, citing the specimen’s unusually short caudal peduncle as a distinctive character. Without examining holotype specimen NMW 22417 and indicating that it had been lost in World War II, Taverne (1971a) initially placed the species under his new genus *Pollimyrus*. Later, recognizing that the name *Heteromormyrus* would have priority over *Pollimyrus*, Taverne (1972) gave *Heteromormyrus* separate generic status. The current collection manager of the Naturhistorisches Museum fish collection, A. Palandačić, cannot confirm that the type was ever missing.

Referring the *Ha*SC species and *P.tavernei* to genus *Heteromormyrus* is one small advance in what will have to be a much larger effort to establish natural (i.e., monophyletic) genera in the family Mormyridae. The sister clade to *Heteromormyrus* in the tree of Peterson et al. (2022) containing the polyphyletic genus *Marcusenius* as well as *Gnathonemus*, *Genyomyrus*, *Cyphomyrus*, and *Campylomormyrus* is in particular need of taxonomic attention. In this case progress was made possible by our ability to recover mitochondrial sequences from a 19^th^ Century holotype specimen, heretofore an unconventional approach. Below we review what we know about the composition of genus *Heteromormyrus*, the morphological characteristics of these species, their geographic distribution and takeaway lessons from this first application of genomic shotgun sequencing to a problem in mormyrid taxonomy.

### Was the *H.pauciradiatus* holotype collected far inland?

Franz Steindachner could not be more specific than “Angola” for the provenance of most of the species treated in his 1866 publication because he had obtained these specimens thirdhand: “all the described species with the exception of the Cyprinoidei I received during my second stay at Cadiz for a not inconsiderable amount of money from a Portuguese merchant who had just returned from Angola” (Steindachner 1866: 771). On maps from the period “Angola” referred to a much smaller area than the modern country of that name, viz. the territory extending east into the interior from Luanda on the coast, sandwiched roughly between the Congo territory north of the Rio Dande and the Benguella territory south of the Cuvo River (Baynes 1878). This region contains the central and lower reaches of the Kwanza, its main northeastern tributary, the Lucala, and the much smaller Bengo River to the north that empties separately into the Atlantic.

In his 1866 article, the first on Angolan freshwater fishes, Steindachner described eight other species (no other Mormyridae), two of which are now regarded as junior synonyms. These taxa—three cichlids, two clariid catfishes, two small barbs and one kneriid—are species characteristic of the Angolan coastal freshwater fauna (Skelton 2019). Given the limited European activity in the interior of this part of Africa at the time (Baynes 1878), we surmised the type of *Heteromormyruspauciradiatus* had been taken near the coast. Hence our surprise to find identical sequences to that of the holotype from specimens collected more than 200 km inland from the upper Lucala River, the Kwanza River’s major northeastern tributary. However, one member of the K4 mitochondrial lineage we now identify as *H.pauciradiatus* was collected in the lower Kwanza, close to the coast. Sampling remains very limited at present and future work may discover mitochondrial haplotypes in the lower Kwanza identical, or nearly so, to that of the type specimen.

### Morphological characteristics of *Heteromormyrus* species

While a complete comparative morphometric and meristic treatment of *Heteromormyrus* species is beyond the scope of this study, for the purposes of preliminary comparisons we have assembled measurements taken by Harder (2000) of relevant mormyrid types (including the *H.pauciradiatus* holotype NMW 22417), measurements and counts in Kramer et al. (2004) and Kramer and Swartz (2010) of *Ha*SC species and forms, and new data taken from photos and radiographs of individuals identified as *H.pauciradiatus* in this study from their mitochondrial DNA sequences (Suppl. material 5).

The unusual appearance of the *Heteromormyruspauciradiatus* holotype NMW 22417 is due to its short dorsal fin (17 rays) placed far back on the body and a very short (but deep) caudal peduncle. This is best captured by the morphometric ratio of the pre-dorsal length (tip of snout to origin of dorsal fin) divided by the standard length (PDL/SL). This ratio is 76% in the holotype, far higher than for any other mormyrid type or non-type specimen included in (Suppl. material 5: table S2). This ratio ranges from 66–70% in the seven measured K4 specimens. In the holotype, caudal peduncle length is only 15% of SL and caudal peduncle depth is 61% of caudal peduncle length, compared to 18–19% and 46–55% respectively in the seven measured K4 specimens now referred to *H.pauciradiatus*.

**Figure 7.**
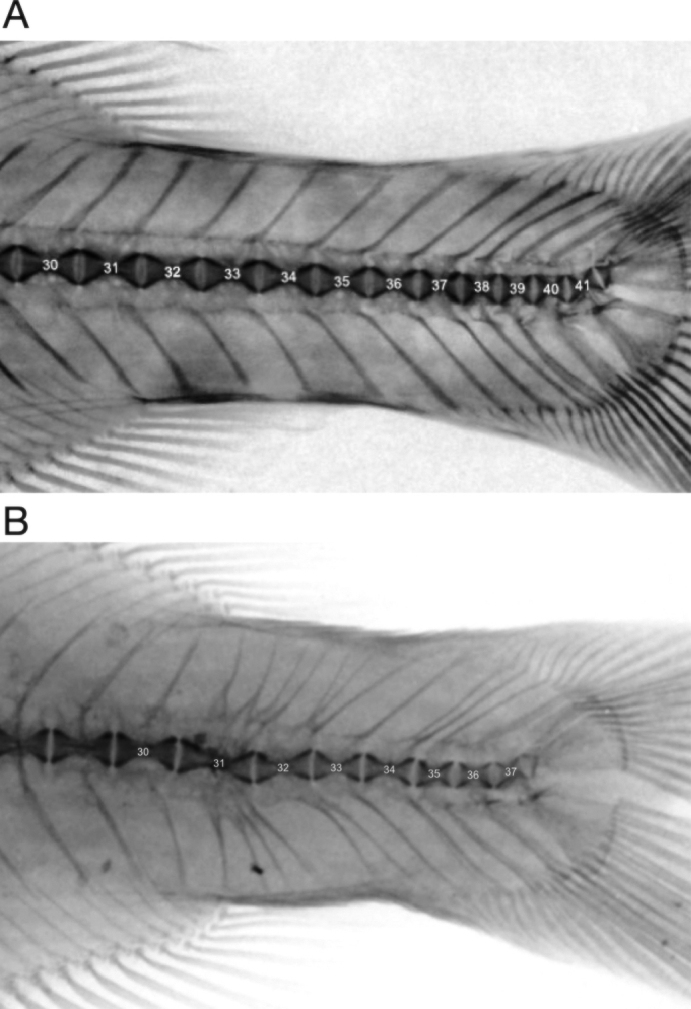
Radiographs showing caudal vertebrae in a recently collected *Heteromormyruspauciradiatus* and in the *H.pauciradiatus* holotype NMW 22417. Vertebrae are numbered from first post-cranial centrum. **A** caudal peduncle of non-type *Heteromormyruspauciradiatus* SAIAB 85120_SAF518-09(originally identified as lineage K4) from the Lucala River of Angola with 41 total vertebrae. Identical vertebral count was found in five other radiographed specimens of this species **B** caudal peduncle of *Heteromormyruspauciradiatus* holotype NMW 22417, showing total vertebral count of 37, four fewer caudal vertebrae than a normal individual, and supernumerary neural and hemal spines at vertebrae 31, 32, and 34.

The reason for the holotype specimen’s strange proportions is revealed in a radiograph showing an abnormality in the caudal skeleton (Fig. 7B). Vertebral centrum 31 in the anterior caudal peduncle is malformed and bears four neural spines and four hemal spines instead of one of each. Centrum 32 in the holotype appears to have three neural spines and two hemal spines. Centrum 33 appears normal with single neural and hemal spines, but centrum 34 bears two neural spines. The last full centrum in the holotype specimen is the 37^th^. Radiographs of five recently collected *H.pauciradiatus* (Fig. 7A, lineage K4 in Mutizwa et al. 2021) all show 41 total vertebrae, with caudal vertebrae bearing a single neural and hemal spine. From what appears to have been a developmental anomaly, the caudal peduncle of NMW 22417 is missing four caudal centra rendering it abnormally short for any kind of mormyrid. This short peduncle gives the specimen an odd appearance and renders all ratio measurements divided by standard length unusually high. The fact that *Heteromormyruspauciradiatus* was defined by an individual with this deformity may help explain why this specific epithet was almost never subsequently used.

Having linked the somewhat misshapen type of *H.pauciradiatus* to recently collected specimens, we can identify some phenotypic characteristics that seem to distinguish this species from its congeners, pending more thorough study. (In the following we remove the holotype from comparisons involving the caudal peduncle.) The body of *H.pauciradiatus* is shorter and deeper and the head is deeper than in other described *Heteromormyrus*: body depth 25–31% of SL, head depth 84–97% of head length. Pre-dorsal distance is a greater percentage of SL than for other *Heteromormyrus*, 66–70%. The caudal peduncle is deeper than in other described *Heteromormyrus*: caudal peduncle depth 46–55% of caudal peduncle length. The eye of this species is small, 12–15% of head length.

As noted above, until now only a single non-type specimen seems to have ever been referred to *H.pauciradiatus*. It is a very large individual (175 mm SL) collected by W.J. Ansorge in the Bengo River at Cabiri (8°54'52"S, 13°39'57"E), identified as *H.pauciradiatus* by George Boulenger (Boulenger 1910), BMNH 1910.11.28.44. The Bengo is a smaller coastal river emptying just to the north of the city of Luanda, Angola and the collection site near Cabiri is not far from the outskirts of the modern city. The specimen has an elongate, wide body with a blunt snout and a very deep caudal peduncle. The morphometrics are sufficiently different from those of the *H.pauciradiatus* holotype and the K4 specimens (Suppl. material 5: table S2) that we think it likely represents a distinct, undescribed species within *Heteromormyrus*, not *H.pauciradiatus*.

The peculiar holotype of *H.pauciradiatus* aside, the rather generalized appearance of all described and undescribed *Heteromormyrus* species and their lack of any obvious shared distinctive phenotypic character(s) may help explain why circumscription of this genus had to wait for molecular phylogenetic analysis. Species of the newly defined genus *Heteromormyrus*, here taken to include *H.pauciradiatus*, *H.ansorgii*, *H.pappenheimi*, *H.tavernei*, *H.szaboi*, *H.longilateralis*, as well as undescribed forms studied in Mutizwa et al. (2021) and the one included here from the Inkisi River are mormyrids of small to intermediate size (< 200 mm SL) with compressed to somewhat wide bodies for mormyrids. Heads are rounded, mouths are terminal to subterminal, chins with less developed swellings than those of species of *Marcusenius*; teeth are notched, usually 7/8 but as few as 5/7 and as many as 9/10. Median fins are situated far back on the body, with the dorsal fin (17–24 rays) origin slightly behind that of the anal (23–27 rays). Scales are 58–72 along lateral line and 12–20 around caudal peduncle. In all species there is a variously developed band of dark pigment on the body between the anterior of the median fins, several scales wide, sometimes broken along the midline. Individuals of several species and undescribed forms also show a blotch of dark pigment on the hypural plate area between the two lobes of the caudal fin.

The study of Peterson et al. (2022) showed the Nilo-Sudanic *Hippopotamyruspictus* (Marcusen, 1864) and *Hippopotamyruspaugyi* Lévêque & Bigorne, 1985 from Upper Guinea to be close relatives of *H.castor*, the type species of genus *Hippopotamyrus*. All three have larger eyes and longer median fins (30 or more rays in both fins) that are equal in length to each other and symmetrically apposed (versus anal origin ahead of dorsal) compared to species and forms of *Heteromormyrus* and so the two genera are in fact not so morphologically similar.

Species of *Heteromormyrus* more closely resemble species of *Brienomyrus* Taverne, 1971, *Paramormyrops* Taverne, Thys van den Audenaerde & Heymer, 1977 and *Ivindomyrus* Taverne & Géry, 1975 in body proportions and counts, hence the mistaken placement of *H.tavernei* originally in *Brienomyrus* and its subsequent transfer to *Paramormyrops* (Rich et al. 2017). These genera are yet more distantly related to *Heteromormyrus* than is *Hippopotamyrus* (Fig. 3; Peterson et al. 2022). Species of *Paramormyrops*, *Ivindomyrus* and *Brienomyrus* lack the strong pigment between dorsal and anal (although some *Paramormyrops* do have very light, diffuse pigment in this location) and the pigment blotch on the hypural region seen in many *Heteromormyrus* species and forms is absent.

It is our hope that targeted study of these similar-appearing clades of mormyrids using computed tomography (CT) will reveal heretofore missed osteological differences and diagnostic characters for some of these genera.

### Newly recognized *Heteromormyrus* species and forms

#### *Heteromormyrusansorgii* (Boulenger, 1905), Fig. 6E

Boulenger (1905a) described *Marcuseniusansorgii* from two specimens collected by W.J. Ansorge from an indefinite location or locations “between Benguella and Bihé,” an area extending more than 400 km from the coast to the interior of what is the modern country of Angola, including the headwaters of several important watersheds (Kramer et al. 2004). However, upon review of Ansorge’s field notes Mutizwa et al. (2021) made the case that these type specimens must have been taken in the Kwanza River basin and so may correspond to one of the five Kwanza lineages (K1–K5) they identified. In his description of *Marcuseniusansorgii* Boulenger noted the similarity of his new species with *H.pauciradiatus* saying “this species is intermediate between *M.lhuysii*, Stdr., and *M.pauciradiatus*, Stdr.” (*Mormyruslhuysii* is today a subjective synonym of *Brevimyrusniger*). Given that the type of *H.pauciradiatus* was a malformed individual, might *H.ansorgii* in fact be a junior synonym of *H.pauciradiatus*? We think not. While Mutizwa et al. (2021) did not explicitly identify *H.ansorgii* with one of the mitochondrial lineages documented in the Kwanza Basin, their morphometric and meristic principal component analyses seem to point to their K3 lineage as the best match and rule out K4 that we herein identify as *H.pauciradiatus*. The *H.ansorgii* syntypes are much more elongate than both the *H.pauciradiatus* type and newly collected specimens, even excluding the post-anal body (Suppl. material 5: table S2), and both the holotype and recently collected examples of *H.pauciradiatus* exhibit a more rounded snout and fusiform body profile than the *H.ansorgii* types and indeed the other described species of *Heteromormyrus*. (The common name “slender stonebasher,” while fitting for *H.ansorgii*, *H.szaboi*, and *H.longilateralis*, would not likely have been applied to *H.pauciradiatus*.)

#### *Heteromormyrusszaboi* (Kramer, van der Bank & Wink, 2004), Fig. 6C *Heteromormyruslongilateralis* (Kramer & Swartz, 2010), Fig. 6D

Quite opposite the example of the unused taxon *Heteromormyruspauciradiatus*, the name *Marcuseniusansorgii* / *Hippopotamyrusansorgii* was initially overapplied to allopatric populations now recognized as multiple species of slender stonebasher (Mutizwa et al. 2021). Work combining morphometrics and meristics, DNA and EOD characters by Bernd Kramer and colleagues showed *H.szaboi* from the upper Zambezi along the Caprivi Strip of Namibia (Kramer et al. 2004) and *H.longilateralis* from the Kunene River at the Namibia/Angola border (Kramer and Swartz 2010) to be distinct species from each other and from the syntypes of *H.ansorgii*.

In the description of *H.szaboi*, additional forms “HaZ” (for Zambezi River) and “HaK” (for Kwando River) were recognized based on EOD and sequence differences from *H.szaboi* but not described as species. The HaZ form was reported to occur in sympatry with *H.szaboi* at its type locality in the Zambezi. More recently, one of us (A.C.) has collected two EOD morphs of a szaboi-like fish in the Kabompo River, a major left-bank tributary of the upper Zambezi in northwestern Zambia. These were included in our phylogenetic analysis of Cyt-*b* sequences which establishes that *H.szaboi* and these *szaboi*-like forms (HaK, HaZ, unrecorded Zambezi fish, and Kabompo EOD morphs 1 and 2) constitute a monophyletic group. This group is divided into two subclades: one consisting of *H.szaboi* sensu stricto and the other containing all the other *szaboi*-like forms. Within the latter subclade there is little structure, despite evident morphological and EOD differences among the specimens (pers. obs.). The species status of these *szaboi*-like forms, referred to as *H.szaboi* in Mutizwa et al. (2021) and here as H.aff.szaboi, remains for future studies to determine.

Research teams from the South African Institute for Aquatic Biodiversity (NRF-SAIAB) have collected other slender stonebashers in southeastern Africa that merit further study and consideration of species status in the Ruo, Buzi, and Pungwe rivers. DNA sequences from these were included in the study of Mutizwa et al. (2021) and in this one.

#### *Heteromormyruspappenheimi* (Boulenger, 1910), Fig. 6F

Boulenger (1910) described this species from W.J. Ansorge’s Angolan collection based on numerous specimens taken at Cunga (Fazenda Cunga / Vila da Cabala on modern maps near the Kwanza River bridge ca. 09°16'54"S, 013°44'48"E). This is the only described species currently placed in *Hippopotamyrus* we are transferring to *Heteromormyrus* without the benefit of sequence data. We know of no other collections of this species since those of Ansorge despite the easy accessibility of the type locality on the lower Kwanza River. This species is something of an outlier morphologically from the others, with a more compressed body, a long, narrow caudal peduncle and higher median fin ray counts than the other species now placed in *Heteromormyrus*. The syntypes today have a bleached appearance but Boulenger’s description indicates the transverse band of pigment between dorsal and anal typical for *Heteromormyrus* species was originally visible. The long peduncle and other differences could be related to its particular habitat in the main channel of lower Kwanza as opposed to the fast flowing, clear-water rocky streams typical of the others. Boulenger comments in his description that this species is “allied to [*Ivindomyrus*] *marchei* and [*Paramormyrops*] *kingsleyae*,” two species from the Ogooué system far to the north. It does bear some similarity to the former of these in particular, another species found in large river channels with a long caudal peduncle. However, no member of the Lower Guinea Clade sensu Sullivan et al. (2016) to which those taxa belong has been recorded south of the Congo Basin. We can be confident it does not belong to genus *Hippopotamyrus* and sequence data from freshly collected specimens will one day test our hypothesis that this species belongs with its Kwanza Basin rivermates in the genus *Heteromormyrus*.

#### *Heteromormyrustavernei* (Poll, 1972), Figs 6B, 8C

From the upper Lualaba and Lufira basins upstream of Lake Upemba in northern Katanga of D.R. Congo, this is the only described species of *Heteromormyrus* from a part of the Congo Basin. Originally placed in genus *Brienomyrus* by Poll (1972), it was recently transferred to genus *Paramormyrops* by Rich et al. (2017), a genus to which many species formerly classified in *Brienomyrus* had been earlier transferred (Hopkins et al. 2007). This species has a distinctively wide head and body with a thick caudal peduncle. The transverse band of pigment between dorsal and anal typical of *Heteromormyrus* species is present although less prominent than in others. Its true affinities were unknown until its inclusion in Peterson et al. (2022) and in this study. The EOD characteristics of *H.tavernei* will be considered in a separate study. This species is currently the subject of doctoral research by C.M.M.

#### *Heteromormyrus* sp. Inkisi River (undescribed), Fig. 8D

Wamuini Lunkayilakio et al. (2010) recorded a fish they called Hippopotamyrusaff.ansorgii from the Inkisi River in D.R. Congo. The Inkisi is a faunistically interesting river that flows due north from Angola, emptying into the Lower Congo River downstream of Pool Malebo. Individuals of this undescribed species were COI barcoded in Sonet et al. (2019). The specimen sequenced for Peterson et al. (2022) and used here comes from a collection by Melanie Stiassny at the American Museum of Natural History in New York. The COI sequenced from this specimen (accession ON84362) is a perfect match to those deposited in GenBank by Sonet et al. (2019). This specimen is a deeper bodied fish, similar to *H.pauciradiatus* in body proportions. Indeed, the COI sequence from it places it as sister lineage to *H.pauciradiatus* (Fig. 5) while Cyt-*b* places it as sister to all *Heteromormyrus* but *H.* sp. K5 (Figs 3, 4). Whichever topology is accurate, we believe it most likely represents an undescribed species distinct from *H.pauciradiatus*.

**Figure 8.**
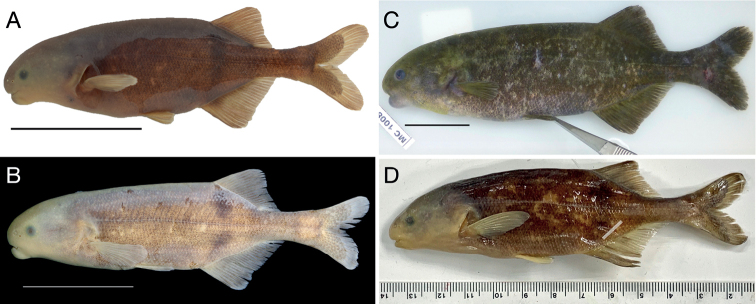
Images of recently collected *Heteromormyrus* specimens from which DNA sequences are used in this study **A***Heteromormyruspauciradiatus* SAIAB 85120_RM, Lucala River above Kalendula Falls, Kwanza Basin, Angola. Partial COI sequence from this specimen is identical to that recovered from *H.pauciradiatus* holotype **B***Heteromormyruspauciradiatus* SAIAB 84683_RM, Lucala River at N’dalatando Farm, Kwanza Basin, Angola **C***Heteromormyrustavernei* RMCA Vert 2018-032-P-0047 tag no. MC-1008, upper Lufira River before the confluence with Panda River, Democratic Republic of the Congo. Specimen sequenced for Peterson et al. (2022) and for this study **D***Heteromormyrus* sp. Inkisi River AMNH 247102, Democratic Republic of the Congo. Specimen sequenced in Peterson et al. (2022) and for this study. Scale bars: 3 cm (**A–C**); millimeter scale (**D**). Photograph credits: SAIAB (**A, B**), J.P.S. (**C**), C.D.H. (**D**).

### Remaining problematic taxa in *Hippopotamyrus*

Even after removing the misclassified *Ha*SC species, genus *Hippopotamyrus* requires more attention as it contains several species that have never been well studied. Below we perform some housekeeping with four species currently classified in this genus, three of which belong neither in *Hippopotamyrus* nor in *Heteromormyrus*. Three of these four determinations lack DNA sequence data and are based on the sum total of available information, including original descriptions and photographs of types available on the Mormyridae Scratchpad website (Sullivan & Lavoué, 2022) and reports from field workers.

#### *Hippopotamyrusharringtoni* (Boulenger, 1905) = junior synonym of *Hippopotamyruspictus* (Marcusen, 1864)

Boulenger’s description of this species (in *Marcusenius*, later placed by some authors in *Gnathonemus* before Taverne (1971a) transferred it to *Hippopotamyrus*) from a single specimen from the Baro River (White Nile) of Ethiopia (Boulenger 1905b) is of dubious status. Lévêque and Bigorne (1985) suggested that the *H.harringtoni* type may simply have been a large specimen of *H.pictus* in which the somewhat more pronounced snout and absence of a lateral band of dark pigment between the dorsal and anal fins are concomitant features of this specimen’s large size (305 mm TL). These authors document the phenomenon of this mark fading in other large specimens of *H.pictus* from West Africa that had been identified as *H.harringtoni*. Their examination of the holotype revealed a faint band between the median fins, similar to the dark one usually seen in *H.pictus*. They reidentified all West African *H.harringtoni* as *H.pictus* but did not formally synonymize the species themselves. Typical *H.pictus* are present in the Baro River of Ethiopia, type locality of *H.harringtoni* (J.P.S.; B. Levin, pers. comm.). We know of no recent specimens from the Baro as large as the *H.harringtoni* type or conforming closely to its phenotype. As more than a century has passed without new specimens corresponding to the description of the *H.harringtoni* type, we think it appropriate to formally place *H.harringtoni* as a junior synonym of *H.pictus*.

#### *Hippopotamyrusmacroterops* (Boulenger, 1920) = *Pollimyrusmacroterops* (Boulenger, 1920), comb. nov.

Boulenger remarks that this species from Poko, Bas Uélé Province in D.R. Congo, described as *Marcuseniusmacroterops*, is “*très voisine*” to *M.tumifrons* Boulenger, 1902 which today is placed in genus *Pollimyrus* Taverne, 1971. With its large eye and dorsal fin terminus farther posterior than the anal fin terminus, it bears even more resemblance in our opinion to *Pollimyrusplagiostoma* (Boulenger, 1898), also from the Congo Basin. There can be little doubt this species belongs in *Pollimyrus*, a genus shown to be monophyletic in Peterson et al. (2022), although this species was not sequenced in that study.

#### *Hippopotamyrusweeksii* (Boulenger, 1902) = *Cyphomyrusweeksii* (Boulenger, 1902), comb. nov.

Another Boulenger species from the Congo Basin described as a *Marcusenius*, later transferred to *Hippopotamyrus* by Taverne (1971a), *H.weeksi* resembles the type species of *Cyphomyrus*, *C.psittacus* (Boulenger, 1897) in having a deep body and a short, blunt snout. However dorsal and anal fins are nearly equal (instead of longer dorsal) and scales are larger than those of other *Cyphomyrus* species. The tree published in Peterson et al. (2022) places a sequenced specimen of this species as the sister to the included *Cyphomyrus* species, rendering *Cyphomyrus* the only sensible placement for it.

#### *Hippopotamyrusgrahami* (Norman, 1928) = *Cyphomyrusgrahami* (Norman, 1928), comb. nov.

This unsequenced species originally described under *Marcusenius* is from the Kagera River and Lake Victoria in eastern Africa. It should be placed in genus *Cyphomyrus* Myers, 1960 by virtue of its arched dorsum and longer dorsal than anal fin. Photographs of specimens from recent collections of this species (D. Twedle, pers. comm.) show it to bear close resemblance to *Cyphomyrusdiscorhynchus* (Peters, 1852).

### Distribution of *Heteromormyrus* species

**Figure 9.**
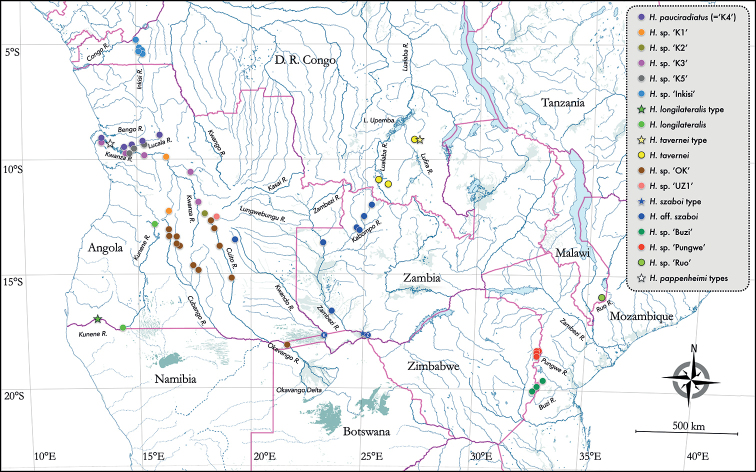
Map of continental Africa between 3° South latitude and Tropic of Capricorn showing distribution of *Heteromormyrus* species and lineages (sequenced specimens and other identified specimens). Type localities shown as star symbols. The localities of two EOD types of H.aff.szaboi, termed HaK and HaZ in Kramer et al. (2004), are indicated by the letters K and Z respectively inside two of the marker dots for H.aff.szaboi. Some points represent multiple adjacent collection sites. Basemap from FaunAfri (Paugy et al. 2008).

The known distribution of *Heteromormyrus* species and populations extends from Atlantic to Indian Ocean watersheds between the latitudes of approximately 5°–20°S, making it the only mormyrid genus restricted to subequatorial Africa (Fig. 9).

The center of diversity for the clade appears to be the Kwanza Basin of Angola in which at least five genetic lineages (K1–K5) occur, some in sympatry (Mutizwa et al. 2021); one of these (K4) we identify here as *H.pauciradiatus*. The type locality of *H.pappenheimi* is on the lower Kwanza and the unspecified type locality of *H.ansorgii* is likely to be somewhere in the upper Kwanza (Mutizwa et al. 2021). While seemingly absent from the central Congo, representatives of this clade occur in opposite southern corners of this basin: the upper Lualaba and Lufira rivers in the east (*H.tavernei*) and the Inkisi River, a left-bank affluent of the lower Congo that flows north from Angola, in the west (undescribed species). The pattern is the opposite that observed for all other mormyrid genera occurring in southern Africa (*Cyphomyrus*, *Marcusenius*, *Mormyrops*, *Mormyrus*, *Petrocephalus*, *Pollimyrus*) that are more diverse in the Congo Basin than in river systems to the south. *Heteromormyrus* species are present in the upper Zambezi Basin (*H.szaboi* and different forms here called H.aff.szaboi) as well as the lower Zambezi (*H.* sp. “Ruo”) but so far absent in collections from the middle Zambezi (Mutizwa et al. 2021).

### Lessons from whole genome shotgun sequencing of a fish holotype specimen

Since the early 1900s the standard practice for preserving fish specimens destined for collections has been fixation in a 10% formalin (= 4% formaldehyde) solution for days or weeks before transfer to 70–75% ethanol or 50% isopropanol. Earlier, fish specimens were simply preserved directly in “spirits,” i.e., an ethanol solution of uncertain concentration. Formalin fixation greatly impairs DNA extraction procedures by cross-linking proteins to the DNA molecules and shearing DNA strands (Zimmermann et al. 2008). Hence 19^th^ Century fish specimens are often the better candidates for DNA extraction and sequencing than those more recently collected, but formalin-fixed.

Sometimes referred to as “museomics,” the application of high throughput sequencing (HTS) to historical DNA (hDNA) from museum specimens (those not originally intended to preserve genetic material and usually less than two centuries old) is emerging as a field distinct from the one concerned with recovery of ancient DNA (aDNA) from much older, naturally preserved samples (Raxworthy and Smith 2021). For hDNA samples, HTS may offer an alternative to the much more time- and labor-intensive Sanger method in which primers must be designed to amplify short stretches of the marker(s) of interest and these fragments separately amplified, sequenced and aligned.

Old fish type specimens have been sequenced before to solve taxonomic puzzles (e.g., Silva et al. 2017) but this may be the first application of whole genome HTS to such a case. Here, employing minimally altered protocols for DNA extraction and Illumina paired-end library preparation we recovered sufficient sequence to use the relatively simple method of “SRA BLASTing” to reconstruct a near-complete mitogenome from a fish holotype preserved more than 150 years ago. We used two markers from the holotype mitogenome to place this taxon in phylogenetic trees produced from sequences of recently collected specimens, resolving the longstanding question of its identity and putting its formerly unshared generic name to good use organizing mormyrid diversity. This approach could be a promising one for countless similar problems across taxonomic ichthyology.

## Supplementary Material

XML Treatment for Heteromormyrus

XML Treatment for Heteromormyrus pauciradiatus

XML Treatment for Heteromormyrus ansorgii

XML Treatment for Heteromormyrus pappenheimi

XML Treatment for Heteromormyrus tavernei

XML Treatment for Heteromormyrus szaboi

XML Treatment for Heteromormyrus longilateralis

## Supplementary materials

Supplementary material 1 List of *Heteromormyrus* specimens examined and/or included in molecular analyses Data type Excel table. https://binary.pensoft.net/file/770296This dataset is made available under the Open Database License (http://opendatacommons.org/licenses/odbl/1.0/). The Open Database License (ODbL) is a license agreement intended to allow users to freely share, modify, and use this Dataset while maintaining this same freedom for others, provided that the original source and author(s) are credited.Author: John P. Sullivan

Supplementary material 2 *Heteromormyruspauciradiatus* holotype NMW 22417 mitogenome reconstruction Data type Text. Explanation note Results of BLASTn search of complete mitogenome of *Hippopotamyruslongilateralis* SAIAB 79793 against SRA archive SRX7700131 of *Heteromormyruspauciradiatus* holotype NMW 22417 and inference of its mitochondrial sequence. https://binary.pensoft.net/file/770297This dataset is made available under the Open Database License (http://opendatacommons.org/licenses/odbl/1.0/). The Open Database License (ODbL) is a license agreement intended to allow users to freely share, modify, and use this Dataset while maintaining this same freedom for others, provided that the original source and author(s) are credited.Author: John P. Sullivan

Supplementary material 3 Cyt *b* plus nuclear markers phylogenetic analysis Data type Image (pdf file). Explanation note Full tree from RAxML analysis with uncollapsed nodes. https://binary.pensoft.net/file/770298This dataset is made available under the Open Database License (http://opendatacommons.org/licenses/odbl/1.0/). The Open Database License (ODbL) is a license agreement intended to allow users to freely share, modify, and use this Dataset while maintaining this same freedom for others, provided that the original source and author(s) are credited.Author: John P. Sullivan

Supplementary material 4 COI phylogenetic analysis Data type Image (pdf file). Explanation note Full tree from RAxML analysis with uncollapsed nodes. https://binary.pensoft.net/file/770299This dataset is made available under the Open Database License (http://opendatacommons.org/licenses/odbl/1.0/). The Open Database License (ODbL) is a license agreement intended to allow users to freely share, modify, and use this Dataset while maintaining this same freedom for others, provided that the original source and author(s) are credited.Author: John P. Sullivan

Supplementary material 5 Measurements and counts of *Heteromormyrus* specimens and relevant mormyrid types from existing literature and taken from photographs & radiographs of newly sequenced individuals Data type Excel table. https://binary.pensoft.net/file/770300This dataset is made available under the Open Database License (http://opendatacommons.org/licenses/odbl/1.0/). The Open Database License (ODbL) is a license agreement intended to allow users to freely share, modify, and use this Dataset while maintaining this same freedom for others, provided that the original source and author(s) are credited.Author: John P. Sullivan
