# Supplementary figures and images for: Mitogenome recovered from a 19 th Century holotype by shotgun sequencing supplies a generic name for an orphaned clade of African weakly electric fishes (Osteoglossomorpha, Mormyridae)
Source: Zookeys. 2022 Nov 16;1129:163–96. doi: 10.3897/zookeys.1129.90287 (PMC9836601; doi:10.3897/zookeys.1129.90287)

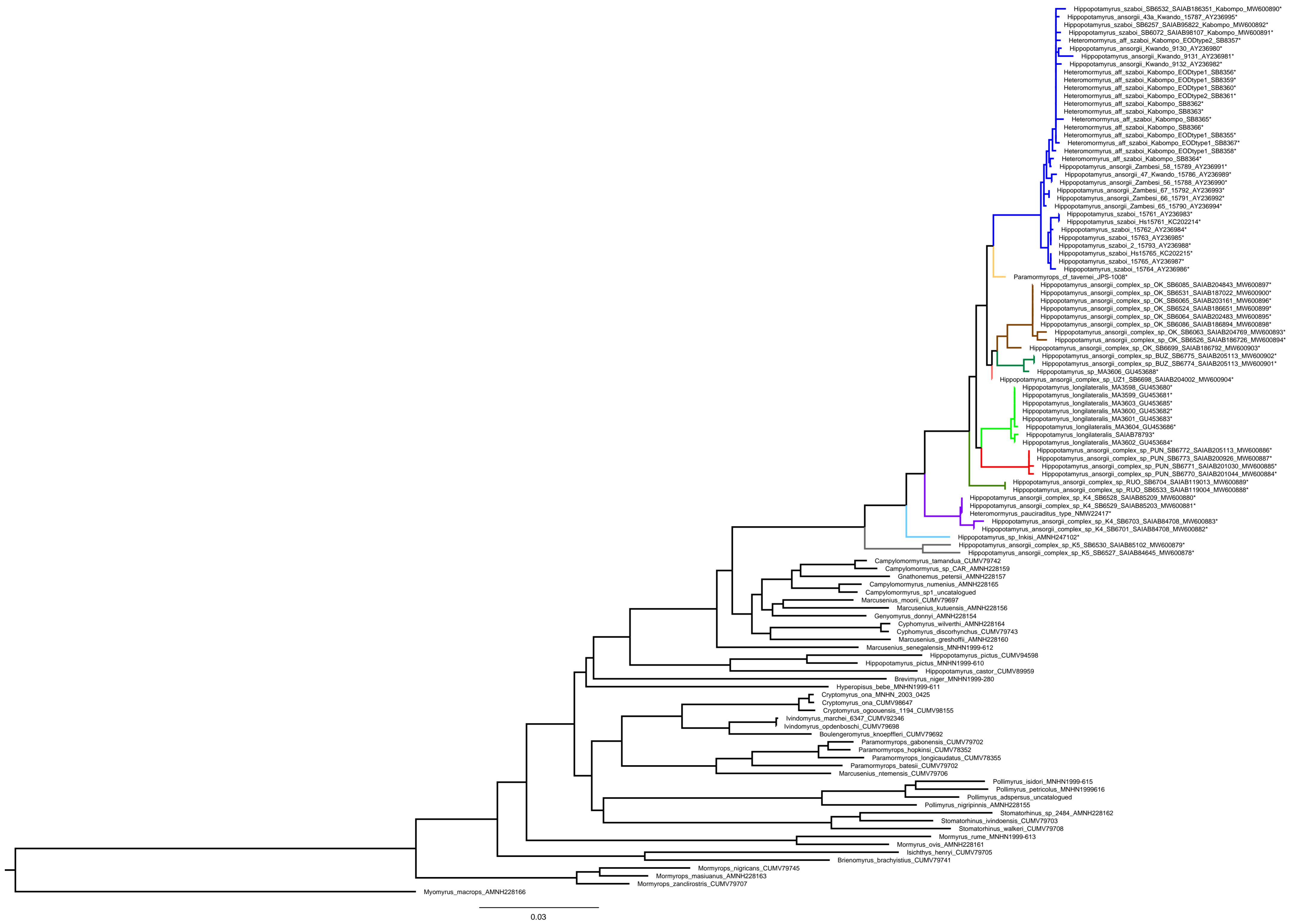

Supplement: Supplementary material 3 — Cyt b plus nuclear markers phylogenetic analysis [file zookeys-1129-163_article-90287__-s003.pdf]

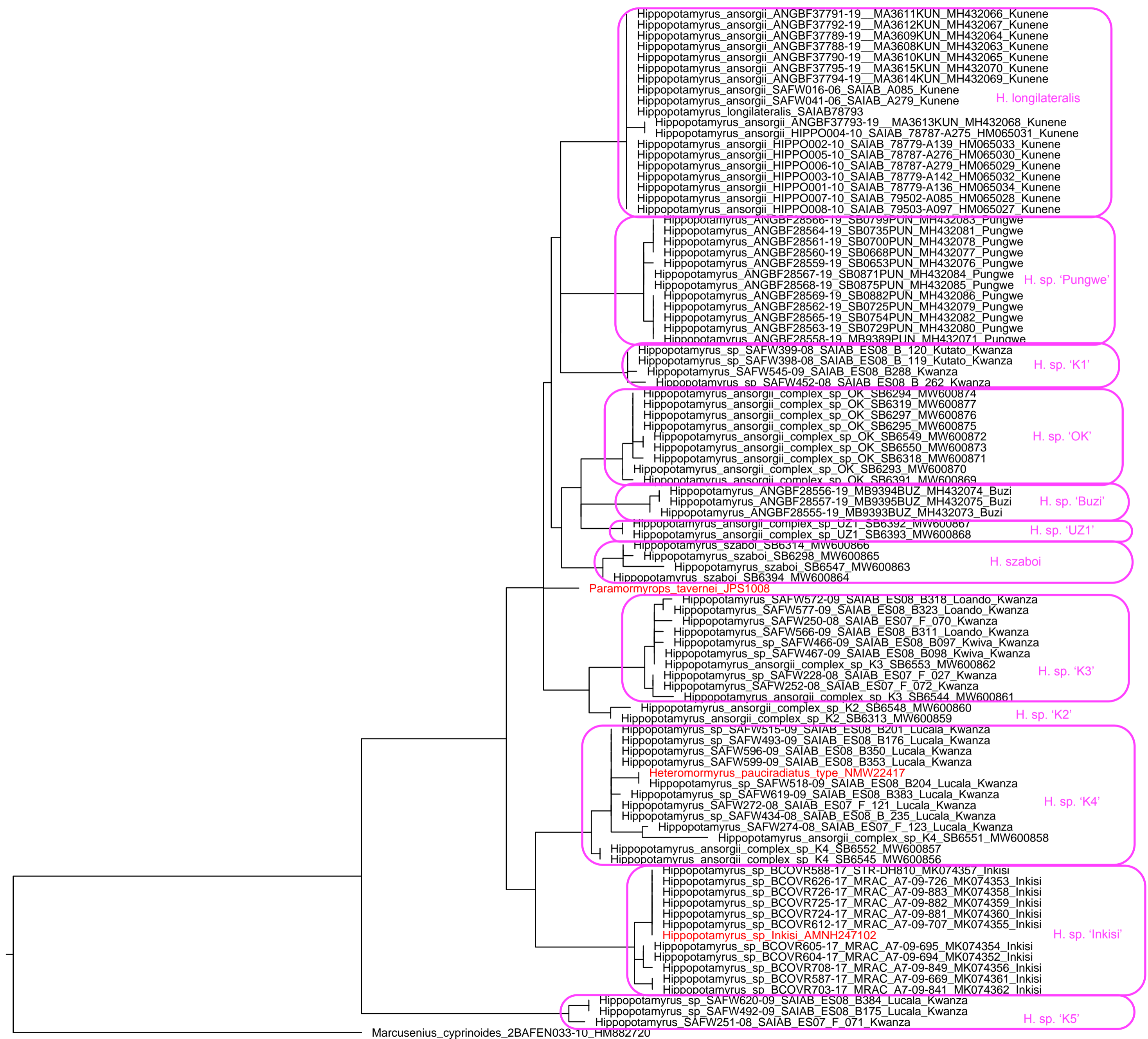

0.04

Supplement: Supplementary material 4 — COI phylogenetic analysis [file zookeys-1129-163_article-90287__-s004.pdf]
